# Antiulcer properties of *Glycyrrhiza glabra L. *extract on experimental models of gastric ulcer in mice

**Published:** 2015

**Authors:** Ghader Jalilzadeh-Amin, Vahid Najarnezhad, Ehsan Anassori, Mostafa Mostafavi, Hadi Keshipour

**Affiliations:** a *Department of Clinical Sciences, Faculty of Veterinary Medicine, Urmia University, Urmia, Iran. *; b *Graduated, School of Veterinary Medicine, Urmia University, Urmia, Iran. *; c *DVM student, School of Veterinary Medicine, Urmia University, Urmia, Iran.*

**Keywords:** Peptic Ulcer, Stress, Ethanol, Indomethacin, *Glycyrrhiza glabra* L

## Abstract

*Glycyrrhiza glabra* L. is used in folk medicine for treatment of stomach disorders including peptic ulcers. The hydroalcoholic extract of *Glycyrrhiza glabra* L. (HEGG) was evaluated for antiulcerogenic activity and acute toxicity profile in mice. Various doses of HEGG (50-200 mg/kg) were administered orally to animals of different groups. Omeprazole and cimetidine at doses of 30 and 100 mg/kg were used as positive controls, respectively. Stomach was opened along the greater curvature then ulceration index was determined examining the inner lining of stomach.

Oral administration of the extract at 1600 mg/kg did not produce toxic symptoms and mortality in mice. 2950 mg/kg was determined as the oral LD_50_. The HEGG (50–200 mg/kg) showed a significant reduction in ulcer index in HCl/Ethanol-induced ulcer. *G. glabra* extract (50-150 mg/kg) showed antiulcer activity against indomethacin-induced gastric lesions dose dependently. The extract effectively inhibited formation of gastric lesions induced by ethanol. The extract (200 mg/kg) was more potent than omeprazole (30 mg/kg). HEGG reduced the ulcer index in hypothermic stress induced gastric ulcers in mice and the antiulcer effect was comparable to that of cimetidine.

The results indicated that *G. glabra* hydroalcoholic extract exerted an antiulcergenic effect that could be associated with increase in gastric mucosal defensive factors.

## Introduction

The genus *glycyrrhiza* consists of approximately 30 species, in which six species produce a sweet saponin glycyrrhizic acid (GA), that is widely used in the confectionery and pharmaceutical industries in various countries (1). Licorice, the roots and stolons of *Glycyrrhiza* species is one of the oldest medicinal plants used for treatment of peptic ulcer (2). Flavonoids (liquritin, glabrol), isoflavones (glabrene, glabridin), chalcones (isoliquiritin), coumarines (liquocoumarin) and stilbenoides besides minor amounts of essential oil and polysaccharides are compounds detected in liquorice root (3). Several studies have indicated that *Glycyrrhiza glabra* extract or its derivatives, mainly glycyrrhizin, bear expectorant, diuretic, laxative, sedative, antipyretic, antimicrobial and anxiolytic, antiviral, anti-inflammatory and antioxidant activities (4).

Gastric and duodenal peptic ulcers affect thousands of people and in the clinical practice are the most common gastrointestinal disorders. Although the etiology of gastric ulcers is still debated, it is accepted that ulcers are caused due to imbalances in mucosal aggressive and self-protective factors. Its pathogenesis is influenced by acid-pepsin secretion, cellular regeneration, mucus secretion, blood flow, mucosal barrier, prostaglandins, epidermal growth (5) and *Helicobacter pylori* infection (6). Particularly, treatment options include antacids, sucralfate, prostaglandins, muscarinic and histaminic antagonists and proton pump inhibitors. In addition, some of these treatments may cause side effects like hypersensitivity, arrhythmia, weakness, and hematopoietic disorders (7). Therefore, search for natural active and better alternative for the treatment of peptic ulcer with fewer side effects is an emergency need. To overcome the drawbacks, many experimental investigations (2, 3, 5, 7) have been undertaken to detect and develop ulcer healing agents by various plant origin compounds. Consequently, the objective of the present study was to validate the antiulcer effect attributed to *G. glabra.* Therefore the antiulcer activity of hydroalcoholic extract of *G. glabra* on four experimental models of acute gastric lesions in mice was investigated.

## Experimental

*Extract preparation*

The plant *G. glabra* L. was identified at the Department of Botany, Faculty of Agriculture, Urmia University, Iran. A voucher specimen (number 6075) was kept in the herbarium. The dried roots and rhizomes were crushed and extracted with 70% v/v ethanol using Soxhlet’s apparatus. The hydroalcoholic extract of *G. glabra* (HEGG) was pooled and concentrated under reduced pressure and evaporated in air to dry. The extract was stored in refrigerator and reconstituted in water for injection.

*Animals*

The study was conducted on Swiss mice of either sex, weighing 25–30 g. The mice were purchased from animal house of Faculty of Veterinary Medicine, Urmia University. The animals were kept under 12 h light-dark cycle and received water and commercial food *ad libitum*. They were fasted for 18 h and then acclimatized to the test environment for 2 h prior to each experiment. The animals were randomly assigned to different groups. Prior to the biological experimentation on animals, the protocols were approved by the Institutional Animals Ethical Committee of the Faculty of Veterinary Medicine, Urmia University.

*Chemicals and solvents*

Cimetidine, omeprazole, and indomethacin were purchased from Sigma (Sigma Chemical Co., St. Louis, USA) and, absolute ethanol, NaCo_3_ and HCL were purchased from Merck (Merck., Germany). All chemicals and extract doses were prepared immediately before use. All the treatments were administered as oral aqueous suspension and the animals of the control group received saline as vehicle.

*Acute oral toxicity and LD*_50_ *determination test*

To evaluate the toxic effect of HEGG different doses (10, 100, 1000, 1600, 2900 and 5000 mg/kg, p. o.) were administrated orally. The general signs of toxicity (i.e., convulsions, ataxia, hypoactivity, ventilation disorders) and mortality were recorded hourly in first day and subsequently all mice were observed twice daily for 14 days after dosing, and then euthanized. The LD_50_ for the HEGG was determined using the method described by Lorke (8). Based on the results, the doses of further pharmacological studies were fixed to be 50, 100, 150 mg/kg and 200 mg/kg orally.

*HCl/Ethanol-induced ulcer*

The HEGG antiulcerogenic activities were assessed based on a method described by Schmeda-Hirschmann *et al. *(7c). Mice were randomly divided into six groups, which fasted for 18 h prior to oral dosing with the saline (10 mL/kg), omeprazole (30 mg/kg) as negative and positive controls, respectively. Remaining groups received HEGG (50, 100, 150 and 200 mg/kg). One hour after the treatments, all animals received 0.2 mL of a 0.3MHCl/60% Ethanol solution orally. Animals were euthanized by cervical dislocation 1 h after the administration of HCl/Ethanol solution; the stomachs were removed and opened along the greater curvature. Gastric contents and blood clots were removed then the stomach rinsed in formaldehyde and fixed between two glass plates. The gastric mucosa was examined for lesions with a binocular stereomicroscope (Nikon SMZ-10).

Ulcer index was calculated following the method described previously (9). The number and severity of lesions were evaluated. The following scores were used: light (I), presence of edema, hyperemia and single petechiae; moderate (II), presence of submucosal hemorrhagic lesions with small erosions; severe (III), presence of hemorrhagic lesions with severe erosions.

Ulcer Index (UI) = (nI) + (nII) 2 + (nIII) 3\Number of animals

Where: n is the number of lesions.

The preventive effect was calculated by the method of Basile *et al.* (10) as follow: 

Prevention index (%) = UI _Control_ – UI _Treated_\UI _Control _× 100

 UI _Control_ = ulcer index in the negative control group 

 UI _Treated_ = ulcer index in the group receiving HEGG

*Indomethacin-induced ulcer*

The HEGG antiulcerogenic activities were assessed in six groups, according to Basile *et al. *(10), modified. Group I (control) received vehicle (5% NaCo_3_) groups 2-5 were pretreated with HEGG 50, 100, 150 and 200 mg/kg *p.o.* respectively, while group 6 received cimetidine (100 mg/kg). One hour after treatment, all the rats received indomethacin (60 mg/kg p.o dissolved in 5% NaCo_3_) to induce gastric ulcer. All the administrations were delivered with oral gavages via the aid of an orogastric cannula. Four hours after indomethacin administration, animals were euthanized by cervical dislocation. The stomachs were removed and opened along the greater curvature.

The tissues were fixed with 10% formaldehyde in saline. Microscopic examination was carried out with a stereomicroscope. Ulcer index (UI) and Preventive Index (PI) of each of the groups was calculated using standard methods as described above.

*Ethanol-induced gastric ulcer*

According to the method of Cerqueira *et al.* (11) acute gastric lesions were induced by the intragastric application of absolute ethanol. Swiss mice were randomly divided into six groups, fasted before the experiment, but had free access to water. Absolute ethanol (0.2 ml/animal, p.o.) was administrated orally to mice, 60 min after the vehicle (saline, controls) or HEGG (50, 100, 150 and 200 mg/kg), while omeprazole (30 mg/kg, p.o.) was used as the reference drug. All treatments were performed by gavage. Thirty minutes after the administration of ethanol, the mice were killed by cervical dislocation, and the stomach was removed and opened along the greater curvature. The stomachs were gently rinsed with water to remove the gastric contents and blood clots, for subsequent examination. Ulcer index and preventive index of each of the groups was calculated using standard methods as described above.

*Hypothermic restraint stress ulcer*

The experiment was performed by the method of Sairam *et al. *(12), with some modifications. After 18 h of starvation, the animals received an oral administration of HEGG (50, 100, 150 and 200 mg/kg), cimetidine (100 mg/kg) or saline (10 mL/kg). One hour after treatment, mice were immobilized in a restraint plastic cage at 4°C for 4 h to induce gastric ulcer. The animals were euthanized and the stomachs were removed and opened along the greater curvature. Ulcer index and preventive index was calculated following the method as described above.

*Statistical analysis*

The experimental data were tested for statistical significance by means of analysis of variance with one-way classification. Sequential differences among means were calculated at a level of p < 0.05 using Tukey contrast analysis. The LD_50_ was determined using Probit Analysis and Maximum Likelihood method with MINITAB software.

## Results and Discussion

*Acute oral toxicity*

It was observed that the hydroalcoholic extract of *G. glabra* (2900-5000 mg/kg, p.o.) induced hypoactivity, mild depression and ataxia in mice of both sexes during the first 30 min and for a period of up to 6 h after administration. However, in the animals treated with dose lower than 1600 mg/kg, it produced no signs of acute toxicity or death. There were no significant changes in food and water intake and or body weight during the 14 days of observation (data not shown). The LD_50_ was estimated about 2950 mg/kg when administered orally in mice.

*Effect of HEGG on HCl/Ethanol-induced ulcer*

Higher doses of hydroalcoholic extract of *G. glabra* and omeprazole showed a similar reduction in ulcer index when compared to the control. The ulcer index was 15.33 ± 0.19 in the control group, while the ulcer index in groups treated with lower doses showed significantly (P < 0.0001) inhibitory effect. Omeprazole was most effective; it reduced the ulcer index to 10.23 ± 0.78 and administration of HEGG with high doses (150-200 mg/kg) showed significant (P < 0.0001) preventive effect in same range of a standard drug used as gastric ulcer inhibition (Table 1).

**Table 1:** The effects of different doses of HEGG (Hydroalcoholic extract of *G. glabra)* on the *HCl/Ethanol-induced ulcer.*

**Groups**	**Dose(** **mg/kg)**	**Ulcer index**	**Preventive Index ** **(%)**
Control	-	15.33±0.19	-
Omeprazole	30	10.23±0.78∗∗	33.26
HEGG	50	13.62±0.25∗∗	11.15
HEGG	100	14.52±0.67∗	5.28
HEGG	150	10.09±0.38∗∗	34.18
HEGG	200	10.33±0.28∗∗	32.61

Results represent data as means ± S.E.M. of six mice in each group.

Significantly different compared to control

(∗ *P* < 0.01,

∗∗ *P* < 0.0001, Tukey test)

*Effect on indomethacin induced gastric ulcers*

Oral treatment with indomethacin developed considerable ulcers in the glandular portion of stomach. Cimetidine (100 mg/kg, p.o) showed a significant reduction in ulcer index (P < 0.0001) when compared with control. Different doses of HEGG (50-150 mg/kg) dose dependently prevented ulcer formation. But HEGG in highest dose (200 mg/kg) show significant and slight effect on indomethacin induced gastric ulcers in comparison with other doses (Table 2).

**Table 2:** The effects of different doses of HEGG (Hydroalcoholic extract of *G. glabra)* on the indomethacin induced gastric ulcers

Groups	Dose(mg/kg)	Ulcer index	Preventive Index (%)
Control	-	18.54±0.14	-
Cimetidine	100	8.35±0.63∗∗	54.96
HEGG	50	16.70±0.11∗∗	9.92
HEGG	100	15.66±0.47∗∗	15.53
HEGG	150	15.01±0.38∗∗	19.03
HEGG	200	17.66±0.40∗	4.74

Results represent data as means ± S.E.M. of six mice in each group.

Significantly different compared to control (

∗ *P* < 0.001,

∗∗ *P* < 0.0001, Tukey test)

*Effects on Ethanol-induced gastric ulcer*

Oral administration of absolute ethanol produced multiple mucosal lesions in the mice stomach. Ulcer indices and preventive indices are shown in Table 3. Pre-treatment with omeprazole and HEGG were found to decrease the ethanol-induced gastric mucosal injuries. Preventive indices of 50, 100, 150 and 200 mg/kg HEGG were in a dose-dependent manner and there was a significant difference between the groups that received different HEGG (P < 0.0001). Omeprazole also significantly inhibited the ethanol-induced gastric lesion (51.2%) compared to that of the control group. There were significant differences between all concentrations of HEGG effects and omeprazole effect (P < 0.0001).

**Table 3:** The effects of different doses of HEGG (Hydroalcoholic extract of *G. glabra)* and omeprazole on the ethanol induced gastric ulcers

Groups	Dose(mg/kg)	Ulcer index	Preventive Index (%)
Control	-	27.50±0.15	-
Omeprazole	30	13.43±0.33∗	51.2
HEGG	50	13.38±0.20∗	51.3
HEGG	100	13.83±0.63∗	49.7
HEGG	150	12.98±0.23∗	52.8
HEGG	200	12.30±0.45∗	55.3

Results represent data as means ± S.E.M. of six mice in each group.

∗ Significantly different compared to control (*P* < 0.0001, Tukey test).

*Effect on Hypothermic restraint stress ulcer*

The oral administration of HEGG at doses of 100, 150 and 200 mg/kg reduced the gastric ulcer indices to 15.66 ± 0.17, 8.41 ± 0.64 and 4.15 ± 0.33, respectively, compared to the control group (32.28 ± 0.97). The HEGG-induced gastroprotection in higher and lower doses were more effective. At similar dose of cimetidine (100 mg/kg) as the standard drug, the HEGG significantly (P < 0.0001) decreased the ulcer index (Table 4).

**Table 4:** The effects of different doses of HEGG (Hydroalcoholic extract of *G. glabra)* on the hypothermic stress induced gastric ulcers

Groups	Dose(mg/kg)	Ulcer index	Preventive Index (%)
Control	-	32.28±0.97	-
Cimetidine	100	18.33±0.45∗	43.21
HEGG	50	26.50±0.11∗	17.90
HEGG	100	15.66±0.17∗	51.48
HEGG	150	8.41±0.64∗	73.94
HEGG	200	4.15±0.33∗	87.14

Results represent data as means ± S.E.M. of six mice in each group.

∗ Significantly different compared to control (*P* < 0.0001, Tukey test).

In this study, the antiulcer effect of Hydroalcoholic extract of *G. glabra* (HEGG) was investigated in mice using stress, ethanol, indomethacin and HCl/Ethanol -induced ulcer models. In addition, the acute toxicity and LD_50_ were evaluated. HEGG inhibit formation of ulcers in all four models and at all doses significantly. The antiulcer capacity of HEGG was determined to be dose dependent. 150-200 mg/kg doses of HEGG inhibited the ulcers more significantly than did omeprazole and cimetidine. The findings that omeprazole and cimetidine orally protected the gastric mucosa against ethanol-induced and stress induced damage are in agreement with the works of other authors (13).

Currently, effective drugs exist for peptic ulcer diseases. However they are very expensive and have multiple side effects that limit their usage. Researchers are now focused on antiulcer agents that are less expensive, less toxic and very effective. Medicinal plants are among the most attractive source of new drugs with hopeful results in peptic ulcer management (14). Besides, being a popular food additive, licorice is also one of the most widely used medicinal herbs in Iran. Present study has evaluated the antiulcerogenic activity of Hydroalcoholic extract of licorice that grows in Iran.

*G. glabra* is a plant that has broad popular use. However, studies are needed to prove the safety of its use, as well as the analysis of acute toxicity is fundamentally important to identify the doses that could be used, and to reveal the possible clinical signs caused by the extract. In investigation of oral acute toxicity of HEGG, mild behavioral alterations and no mortality were observed in doses of 2900 mg/kg, indicating low toxicity of the extract. It was observed that HEGG has LD_50_ = 2950 mg/kg. According to Morrison *et al.* (15), substances that present LD_50_ between 500-5000 mg/kg by oral route can be considered practically slightly toxic. The data obtained in the acute toxicity test suggest that the toxic dose of HEGG (p.o.) in the acute oral toxicity test by the Lorke procedure (8) is higher than 2,900 mg/kg. Previous studies did not show that the LD_50_ of the HEGG in mice to verifying our results. The acute toxicities of licorice extract and glycyrrhizin salts are low with oral LD_50_ generally greater than 4 g glycyrrhizinate/kg b.w. in mice and rats (16). It should be emphasized that the doses that displayed antiulcer activity are much lower than the LD_50_.

Gastric ulcers induced with ethanol and ethanol/HCl are the most commonly utilized experimental models for the evaluation of antiulcer activity in mice (17). The creation of ethanol-induced gastric lesions is of multifactorial origin with decrease in gastric mucus amount as one of the involved factors. A depletion of stomach wall mucus after oral ethanol administration has also been reported (18). Submucosal venules constriction lead to stasis of blood flow in mucosal microcirculation, subsequently cause plasma leakage from the vascular bed, contributing to the widespread mucosal injury in this model. The formation of gastric mucosal lesions by necrotizing agents such as HCl and ethanol has been reported to involve the depletion of gastric defensive mechanisms, the production of mucus and bicarbonate secretion (19). Ethanol and HCl are corrosive agents that damage the gastric mucosa. The HEGG was effective in this model and this effect may be due to increased mucus secretion that protects gastric mucosa from corrosive effects of ethanol.

Indomethacin induced gastric ulcers is used to study the role of prostaglandins on gastric cytoprotection. Indomethacin a non-steroidal anti-inflammatory agents, decrease cyclooxygenase activity and then production of endogenous prostaglandin levels in the gastric mucosa (20), leading to gastric hypermotility and vascular disturbances, thus stimulating the activation of reactive oxygen species (ROS) production, lipid peroxidation and infiltration of neutrophils (21). Endogenous prostaglandins regulate mucosal blood flow, epithelial cell proliferation, epithelial restitution, mucosal immunocyte function, mucus and bicarbonate secretion, and basal acid secretion. Indomethacin inhibits prostaglandin synthesis and this deficiency in prostaglandins is responsible for ulceration (22). HEGG has antiulcer properties and significantly reduced the damage of gastric mucosa induced by indomethacin, suggesting that its gastroprotective effect involves the increase in the prostaglandin synthesis. It seems that by raising the local concentration of prostaglandins that promote mucous secretion and cell proliferation in the stomach, leading to healing of ulcers in experimental studies (23). The possibility of HEGG exerting a cytoprotective effect is indicated because it successfully prevents the formation of gastric lesions in the indomethacin and ethanol induced ulcer models.

Stress is initiated by in cooperation of physiological and psychological factors. This model, cold restraint stress-induced gastric ulcers, has been used due to data reproducibility and similarity with human gastric ulcers to evaluate the effect of drugs on gastric secretion, stress and gastrointestinal motility. Stress increases the formation of ROS, decreases the cell proliferation rate, increases gastric juice secretion, and promotes inhibition of prostaglandin synthesis, leading to alterations in the circulating nitric oxide and the gastric mucosa (24). In the present study, the HEGG protected the gastric mucosa of animals against stress-related injuries, confirming the gastroprotective effect of HEGG.

Licorice root contains triterpenoid saponins (4–20%), mostly glycyrrhizin, a mixture of potassium and calcium salts of glycyrrhizic acid. Anti-ulcer properties of saponins have been reported (25). Principally glycyrrhizin reduces ROS generation which is the potent mediator of tissue inflammation. β-Glycyhrritinic acid is the major metabolite of glycyrrhizin has shown antiinflammatory properties in different animal models (26). Two mechanisms have been suggested includeing inhibition of glucocorticoid metabolism and potentiating their effects, and inhibition of classical complement pathway activation. Glabridin is an isoflavonoid derivatives present in licorice. In *in-vitro* study, the generation of ROS was also suppressed by glabridin (27). Additionally, it was a potent antioxidant toward LDL oxidation in *in-vitro* and *in-vivo* studies. It seems that two mechanisms may be involved in possess of this property by glabridin: first it binds to the LDL and substantially protects its oxidation. Second it accumulates in immune cells, causing a reduction of cellular oxidative stress by reducing NADPH oxidase activation and increasing cellular glutathione (28). Other components of licorice as glyderinine, hispaglabridin A, hispaglabridin B and 4′-O-methylglabridin, showed an antiinflammatory and antioxidant activities (29).

*G. glabra* extracts showed great antioxidant and free radical scavenging activities may be used in order to protect the tissues against damage caused by free radical and ROS (30). The antioxidant properties of flavonoids and tannins have been related to anti-ulcer activity (31) since free radicals are developed in gastric mucosal lesions. Flavonoids have shown cytoprotective activity in various models (32). Recently it was shown that licorice in a combination product could heal ulcers effectively (33) that strongly confirm results of this study.

Thereby it was reported, *G. glabra* contain a number of flavonoids, alkaloids and many other chemical constituents (29). The constituent(s) responsible for antiulcer effect of *G. glabra* are not fully known. Several flavonoids, present in different plants are known to reduce gastric ulcer formation (34). Hence, it is assumed that flavonoids may contribute at least in part to the antiulcer effect of the plants. Apart from flavonoids, the licorice also contains steroids such as beta sitosterol that is known to reduce the development of gastric ulcers (35) and glycyrrhizinic acid that possess gastric antiulcer properties (23). Hence, these constituents may be responsible for reduction of gastric ulcer indices and antiulcer effect of *G. glabra *extract that observed in present study. This also implies that the antiulcergenic activity afforded by HEGG may be associated with its antiinflamatory action, antioxidant properties, cytoprotective activity, and also due to increase in the prostaglandin formation.

 The HEGG protected the stomachs of the mice from induced acute ulceration in various models and did not present acute toxic effects, providing scientific support to the traditional use of this plant. This effect could be related to an increase of gastric mucosal defensive factors. Further pharmacological studies are being undertaken in order to provide more precise elucidation of the action mechanism involved in this activity.
